# Prevalence and Phenotypic Expression of Mutations in the *MYH7, MYBPC3* and *TNNT2* Genes in Families with Hypertrophic Cardiomyopathy in the South of Brazil: A Cross-Sectional Study

**DOI:** 10.5935/abc.20160133

**Published:** 2016-09

**Authors:** Beatriz Piva e Mattos, Fernando Luís Scolari, Marco Antonio Rodrigues Torres, Laura Simon, Valéria Centeno de Freitas, Roberto Giugliani, Úrsula Matte

**Affiliations:** 1 Faculdade de Medicina - Universidade Federal do Rio Grande do Sul, Serviço de Cardiologia - Hospital de Clínicas de Porto Alegre, RS - Brazil; 2 Serviço de Cardiologia - Hospital de Clínicas de Porto Alegre, RS - Brazil; 3 Centro de Terapia Gênica - Hospital de Clínicas de Porto Alegre, RS - Brazil; 4 Instituto de Biociências - Universidade Federal do Rio Grande do Sul, Serviço de Genética Médica - Hospital de Clínicas de Porto Alegre, RS - Brazil; 5 Instituto de Biociências - Universidade Federal do Rio Grande do Sul, Unidade de Análise de Moléculas e Proteínas - Hospital de Clínicas de Porto Alegre, RS - Brazil

**Keywords:** Mutation / genetics, Cardiomyopathy, Hypertrophic, Epidemiology, Sarcomeres, Ethnicity and Health

## Abstract

**Background:**

Mutations in sarcomeric genes are found in 60-70% of individuals with familial forms of hypertrophic cardiomyopathy (HCM). However, this estimate refers to northern hemisphere populations. The molecular-genetic profile of HCM has been subject of few investigations in Brazil, particularly in the south of the country.

**Objective:**

To investigate mutations in the sarcomeric genes *MYH7, MYBPC3* and *TNNT2* in a cohort of HCM patients living in the extreme south of Brazil, and to evaluate genotype-phenotype associations.

**Methods:**

Direct DNA sequencing of all encoding regions of three sarcomeric genes was conducted in 43 consecutive individuals of ten unrelated families.

**Results:**

Mutations for CMH have been found in 25 (58%) patients of seven (70%) of the ten study families. Fourteen (56%) individuals were phenotype-positive. All mutations were missense, four (66%) in *MYH7* and two (33%) in *MYBPC3*. We have not found mutations in the *TNNT2* gene. Mutations in *MYH7* were identified in 20 (47%) patients of six (60%) families. Two of them had not been previously described. Mutations in *MYBPC3* were found in seven (16%) members of two (20%) families. Two (5%) patients showed double heterozygosis for both genes. The mutations affected different domains of encoded proteins and led to variable phenotypic expression. A family history of HCM was identified in all genotype-positive individuals.

**Conclusions:**

In this first genetic-molecular analysis carried out in the south of Brazil, we found mutations in the sarcomeric genes *MYH7* and *MYBPC3* in 58% of individuals. *MYH7*-related disease was identified in the majority of cases with mutation.

## Introduction

Hypertrophic cardiomyopathy (HCM) is the most prevalent genetic cardiovascular disease, affecting one in every 200 individuals.^1-3^ It is a global disease that occurs in many ethnic groups and in both sexes.^3^ HCM has an autosomal dominant pattern of inheritance, and incomplete, age- and gene-dependent penetrance.^4,5^ More than 1,500 causing mutations have been identified, mostly involving 11 genes that encode sarcomeric and Z-disc proteins.^4-6^ More recently, sarcomere mutations in other cell structures have been detected, although its pathogenicity has not been established.^5,6^ HCM-causing mutations are usually identified in 60-70% of patients with familial disease, and 30-40% of patients with the sporadic form.^2,4,6-10^ The involvement of cardiac β-myosin heavy chain (*MYH7*) and myosin-binding protein C (*MYBPC3*) encoding genes is observed, respectively, in 25% to 35% of cases seen in the northern hemisphere.^4,6,10,11^ Troponin T gene (*TNNT2*) mutations are detected in 5% of patients, and mutations in other genes in a lower frequency (<1%). ^5,6^

The heterogeneous molecular substrate and variable phenotypic expression, characteristics of HCM, may be influenced by ethnic and geographical factors. However, the genetic profile of HCM has been established based on studies conducted mostly on northern hemisphere populations,^7-9,12-30^ whereas few similar studies have been carried out in the southern hemisphere.^31-34^ Compared with other countries, the genetic structure of Brazilian population is defined by a high degree of admixture between European, African and Indian ancestors.^35^ Based on genetic aspects peculiar to the south of Brazil, characterized by a low degree of admixture among individuals with different ancestry, we have an interest in defining the genetic profile of HCM in this region.^36^

The aim of this study was to investigate mutations on the sarcomere genes *MYH7, MYBPC3* and *TNNT2* and genotype-phenotype associations in a cohort of HCM patients in the extreme south of Brazil.

## Methods

### Selection of patients and clinical evaluation

A cross-sectional study was conducted on a convenience sample of 43 consecutive individuals from 10 unrelated families, registered in the HCM outpatient care setting of a tertiary hospital in the south of Brazil. The first-degree relatives who first volunteered to participate during the recruitment period were enrolled in the study. All participants were from this region of the country.

The phenotype was defined by the identification of asymmetric left ventricular hypertrophy (LVH) in the echocardiogram, expressed by a maximum wall thickness ≥ 15 mm in any segment with a posterior septum/wall ratio ≥ 1.3, in the absence of chamber dilation or other conditions that may indicate similar changes. A maximum left ventricular (LV) wall thickness ≥ 13 mm in the anterior septal was the criterion used for the identification of HCM in the relatives. All subjects underwent cardiovascular assessment by resting electrocardiogram and echocardiogram. Ten patients underwent coronary angiography. The study protocol was approved by the local Ethics Committee, and signed informed consent was obtained from all participants.

### Molecular-Genetic analysis

DNA was extracted from the peripheral blood according to the technique described by Miller et al.^37^ Amplificatons of all the enconding regions of the sarcomeric genes *MYH7* (38 exons), *MYBPC3* (33 exons) and *TNNT2* (15 exons) was performed by PCR,^38^ by using oligonucleotides available at htpp://www.cardiogenomics.org. The fragments were purified by Exo-SAP, according to the manufacturer's instructions (USB Corporation, USA), followed by direct sequencing of the fragments using BigDye Terminator v3.1 Cycle Sequencing Kit (Applied Biosystems, USA) and capillary electrophoresis using the ABI 3500 Genetic Analyzer (Applied Biosystems, USA). The resulting sequences were then compared with the reference sequences *MYH7* - NM_000257, NP_000248; *MYBPC3* - NM_000256, NP_000247; *TNNT2* - NM_000364, NP_000355. The nomenclature for the description of sequence variants was established by following the *Human Genome Variation Society* recommendations.^39^ In some cases, analyses of cosegregation of the mutation and clinical data were conducted for pathogenicity definition.

*In silico* analysis was used to evaluate the effect of an aminoacid substitution based on the conservation of the regions affected, using the PolyPhen2,^40^ SIFT,^41^ PROVEAN,^42^ MutationTaster,^43^ and MutPred^44^ bioinformatic tools. The MutPred system was used to formulate hypothesis on structural and functional properties of mutation. Synonymous mutations and substitutions in introns and coding exons, neither reported as polymorphisms (SNPs) nor found on the *1000 Exome Variant Server (EVS) database* were also evaluated by *in silico* analysis to identify potential splice site changes. NetGene2^45^ and Human Splicing Finder^46^ were used to calculate the consensus values of potential splice sites.

### Statistical analysis

Quantitative data were expressed as mean and standard deviation, and categorical variables as relative and absolute frequencies. The Shapiro-Wilk test was used to test normality of data, and differences between two groups, based on continuous and symmetrical variables were tested by Student's t-test for independent samples. The categorical variables were compared by the chi-square test. Analyses were performed using the SPSS software*,* version 18.0 (*SPSS Inc., Chicago Illinois, USA*). Significance level was set at p <0.05.

## Results

### Clinical and molecular-genetic profile

The study group was composed of 10 consecutive probands from unrelated families and 33 first-degree relatives. All participants were Caucasians. Clinical characteristics of the cohort are presented in Table 1.

**Table 1:** Clinical characteristics of a cohort of patients with hypertrophic cardiomyopathy in the south of Brazil, composed of 10 unrelated probands and 33 relatives

Characteristics	Probands (n = 10)	Relatives (n = 33)	
		Phenotype-positive (n = 7)	Phenotype-negative (n = 26)
Age (years)	53 ± 7	42 ± 20	32 ± 17
**Racial group**			
Caucasians	10 (100%)	7 (100%)	26 (100%)
Female	5 (50%)	4 (57%)	17 (65%)
**Family history**			
HCM	7 (70%)	7 (100%)	15 (58%)
Sudden cardiac death	2 (20%)	4 (57%)	8 (30%)
Age at the onset of disease (years)	44 ± 12	39 ± 20	-
**NYHA functional class**			
I/II	5 (50%)	5 (71%)	-
III/IV	5 (50%)	2 (29%)	-
**Echocardiogram**			
Left atrial diameter (mm)	46 ± 5	38 ± 8	32 ± 7
LV end-diastolic diameter (mm)	43 ± 5	47 ± 5	45 ± 4
LV end-systolic diameter (mm)	25 ± 3	26 ± 3	25 ± 3
LV maximal parietal thickness (mm)	20 ± 4	20 ± 5	9 ± 7
Ejection fraction %	72 ± 6	74 ± 5	71 ± 5
LV outflow tract obstruction	7 (70%)	-	-
LV mid-ventricular obstruction	1 (10%)	2 (29%)	-
LV outflow gradient (mmHg)	45 ± 33	-	-
**Treatment**			
Alcohol septal ablation	3 (30%)	-	-
Myectomy	1 (10%)	-	-
Double-chamber pacemaker	4 (40%)	-	-
Implantable cardioverter defibrillator	2 (20%)	1 (14%)	-

Data expressed in mean ± standard deviation; HCM: hypertrophic cardiomyopathy; NYHA: New York Heart Association; LV: left ventricular.

HCM-causing mutations were detected in 25 (58%) subjects, 7 (70%) probands and 18 (54%) relatives, from 7 (70%) out of 10 study families. In the families with known mutations, mutations were detected in 18 (82%) of 22 relatives, but only 7 of them (32%) were classified as phenotype-positive. All phenotype-negative relatives (n=15;45%) were normal at clinical examination. In all 11 mutation carriers, without evidence of LVH by echocardiogram, abnormal electrocardiogram, including pathological Q-waves ≥ 3 mm and/or > 40 ms in two or more leads, except for aVR (n = 10;90%), fascicular block (n = 6;54%), deep S-waves in V2 > 25 mm (n = 3;27%), and negative T-waves > 3 mm (n = 1;1%) were detected.

**Figure 1:**
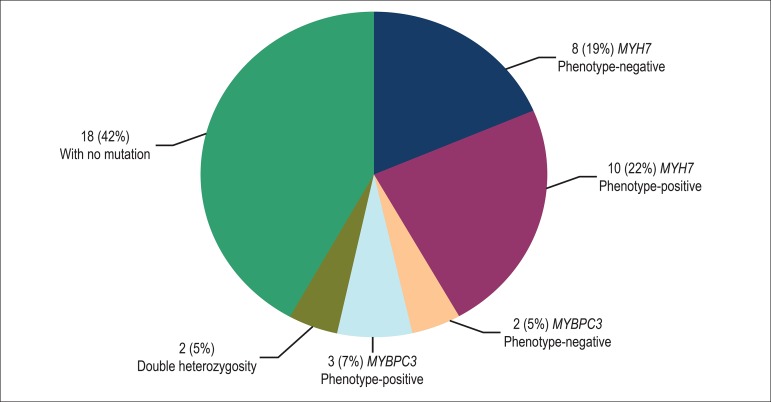
Distribution of mutations in the sarcomeric genes MYH7 and MYBPC3 in a population with hypertrophic cardiomyopathy. Individuals with double heterozygosity were included in this subgroup only.

All mutations identified were missense mutations, four (66%) in the *MYH7* gene and two (33%) in *MYBPC3*. No mutation was detected in *TNNT2*. Two of the four mutations in *MYH7* had not been reported in the literature. Mutations in this gene were identified in 20 (47%) individuals from six (60%) families, including the probands and 14 relatives. Mutations in the *MYBPC3* gene were found in seven (16%) individuals from two (20%) families, including the probands and five relatives. In a single family, two individuals (5%) - the proband and a relative - had double heterozygosis with mutations both in th*e MYH7* and *MYBPC3* genes (Figure 1). In three (30%) genotype-negative families, all members were clinically normal, whereas in the seven families in whom mutations were detected, 32% of the individuals were phenotype-positive. Characteristics of the mutations are described in Table 2 and results of pathogenicity analysis in Table 3.

**Table 2:** Mutations in the sarcomeric genes *MYH7* and *MYPC3* in the study population

Gene/Exon	Mutation/Domain	Change of aminoacid	Families	Number of patients affected (phenotype +/ phenotype -)	Cosegregation	Phenotype
***MYH7***						
9	*Missense/Head*	p.lle263Thr	IX	1/0	NT	Mild, obstructive LVH
21	*Missense/ Domain IQ*	p.Ala797Thr	VI	1/1	NT	Moderate, obstructive LVH
22	*Missense/Neck*	p.Met877lle	V	3/0	Yes	Mild to severe LVH, non-obstructive or with outflow tract obstruction, or mid-ventricular obstruction
32	*Missense/Rod*	p.Glu1468Lys	IV, VIII, X	6/8*	Yes	Mild to severe LVH, non-obstructive or with outflow tract obstruction, or mid-ventricular obstruction, late sudden death
***MYBPC3***						
18	*Missense/C4*	p.Arg495Gln	III	3/2	Yes	Moderate to massive LVH, non-obstructive form of early onset, premature sudden death
25	*Missense/C6*	p.Val896Met	VIII	1/1**	NT	Moderate, obstructive LVH

Domain IQ: calmodulin binding domain; NT: not tested; LVH: left ventricular hypertrophy;

* associated with p.Val896Met (MYBPC3), n = 2;

** associated with p.Glu1468Lys (MYH7), n = 2.

**Table 3:** Analysis of pathogenicity of the mutations in *MYH7* e *MYBPC3* genes

Mutation	PolyPhen2	SIFT	PROVEAN	MutationTaster	MutPred
p.lle263Thr	Benign	Deleterious	Deleterious	Deleterious	Deleterious
p.Ala797Thr	Benign	Benign	Benign	Benign	Deleterious
p.Met877Ile	Benign	Benign	Benign	Deleterious	Benign
p.Glu1468Lys	Deleterious	Deleterious	Deleterious	Deleterious	Deleterious
p.Arg495Gln	Deleterious	Benign	Benign	Deleterious	Deleterious
p.Val896Met	Benign	Deleterious	Benign	Benign	Deleterious

### Mutations in the *MYH7* gene

In the six (60%) families with HCM caused by the *MYH7* gene, only 11 (55%) were phenotype-positive, the proband and five relatives. In the phenotype-positive individuals, the maximal wall thickness varied from 13 to 26 mm (mean of 20 ± 4 mm). In *MYH7*, four mutations were mapped. The substitution p.Ile263Thr was identified in only one family, affecting the proband only. The only relative who underwent genotyping was clinically normal and did not harbor a mutation. The p.Ala797Thr mutation was detected in two members of the same family, the proband and one relative phenotype-negative. This substitution, considered pathogenic exclusively by the MutPred system, has been reported in the *1000 EVS*, with an allele frequency of 0.0002 in African-American population. Both mutations were associated with mild to moderate HCM, and with the obstructive forms of the disease. The p.Met877Ile mutation, a mutation not previously described, was detected in three individuals of a four-member family. The mutation was considered a benign condition by four of the five *in silico* bioinformatic tools used in the study. However, it showed cosegregation with HCM phenotype in HCM patients, indicating a pathogenic effect. The proband had a phenotype of moderate HCM and left ventricular outflow tract obstruction. Severe mid-ventricular obstruction was identified in one family member and mild LVH was detected in another. None of these three mutations were related with a family history of sudden death. The p.Glu1468Lys mutation, identified in three families, had not been previously reported in the literature or in the EVS and SNP databases. Nevertheless, all bioinformatic tools favored its pathogenic potential. Analysis of cosegregation showed that all family members affected were also carriers of mutations, except for one, who was considered normal. In one of the families, although the mutation was identified in the proband and in six of seven members evaluated, only three of them were phenotype-positive. The proband and one of the family members had non-obstructive, moderate HCM. Mid-ventricular obstruction was identified in another family member, who died suddenly at the age of 66 during the study period. In another family with this mutation, the proband and four relatives had this genotype, although only two were phenotype-positive. Two individuals, one proband and one young, phenotype-negative relative, showed a double heterozygosis with mutations both in *MYH7* and *MYBPC3*. The proband had moderate LVH and severe left ventricular outflow tract obstruction, whereas the relative with isolated mutation in *MYH7* had mild, non-obstructive form of the disease. In the third family affected, the proband and one young, phenotype-negative relative were carriers of this mutation, which associated with moderate LVH and severe left ventricular outflow tract obstruction.

### Mutations in the *MYBPC3* gene

In the two (20%) families with mutations in the *MYBPC3* gene, four (57%) of the carriers were phenotype-positive, the proband and two relatives. The mutations led to LVH with marked inter- and intrafamilial variability, expressed by a maximal wall thickness of LV varying from 17 to 34 mm, and mean of 23 ± 7 mm. The mutation p.Arg495Gln was identified in one family, in the proband and in four of the five members who underwent genotyping, two of them phenotype-positive. The analysis of cosegregation in this family and three *in silico* bioinformatic tools favored the pathogenic role of this mutation in HCM. The mutation was related to early onset and to non-obstructive forms with mild to massive LVH associated with a family history of premature sudden death. The mutation p.Val896Met was found in two subjects of a six-member family, who were also carriers of the mutation p.Glu1468Lys in the *MYH7* gene. Two programs indicated the pathogenic effect of this variant, which was also reported in the EVS, with an allele frequency of 0.0015 and 0.0048 in African- and European-origin individuals respectively.

The comparison of clinical variables between genotype-positive and genotype-negative subjects revealed that a family history of HCM and/or sudden death was more frequent in those in whom a mutation was identified (Table 4). Clinical indicators were not significantly different between carriers of mutations of the two genes, except for a lower age and a family history of sudden death, which associated with HCM caused by *MYBPC3* (Table 5).

**Table 4:** Comparison of clinical characteristics between genotype-positive and genotype-negative subjects

Characteristics	Genotype-positive (n = 25)	Genotype-negative (n = 18)	p
Age (years)	41 ± 19	35 ± 17	0.3
**Gender**			
Male	13 (52%)	4 (22%)	0.08
Female	12 (48%)	14 (78%)	
**Family history**			
HCM	25 (100%)	4 (22%)	0.0001
Cardiac sudden death	12 (48%)	2 (11%)	0.019

Data expressed in mean ± standard deviation; HCM: hypertrophic cardiomyopathy.

**Table 5:** Comparison of clinical characteristics of carriers of mutations in the *MYH7* and *MYBPC3* gene

	*MYH7* (n = 20)	*MYBPC3* (n = 7)	p
Age (years)	48 ± 19	32 ± 16	0.102
Age at the onset of disease (years)	47 ± 13	25 ± 13	0.0001
**Gender**			
Male	13 (65%)	5 (71%)	0.127
Female	7 (35%)	2 (29%)	
**Family history**			
HCM	20 (100%)	7 (100%)	-
Sudden death	7 (35%)	5 (71%)	0.016
**NYHA functional class**			
I/II	15 (75%)	6 (86%)	0.246
III/IV	5 (25%)	1 (14%)	
Left atrial diameter (mm)	40 ± 6	33 ± 7	0.082
LV diastolic diameter (mm)	45 ± 5	40 ± 4	0.06
LV systolic diameter (mm)	26 ± 4	24 ± 2	0.195
LV maximal parietal thickness (mm)	15 ± 6	19 ± 10	0.274
Ejection fraction (%)	67 ± 19	70 ± 3	0.497
LV outflow tract obstruction	6 (30%)	1 (14%)	0.133

Data expressed in mean ± standard deviation; HCM: hypertrophic cardiomyopathy; NYHA: New York Heart Association; LV: left ventricular.

## Discussion

The present study represents the first genetic-molecular analysis of HCM conducted on a population from the extreme south of Brazil, composed of members of unrelated families. A sample of consecutive index-patients and their respective relatives, considered representative of a cohort of non-referred HCM outpatients was studied. Causing mutations were detected in 58% of subjects and 70% of the families. Mutations in *MYH7*, identified in 47% of patients, were more frequent than mutations in *MYBPC3,* found in 16% of patients. Most mutations were private. Two mutations in the *MYH7* gene were considered novel. A positive genetic test in the proband enabled the molecular identification of the disease in up to 82% of the respective relatives, 39% of them phenotype-positive. All mutation carriers without evidence of HCM in the echocardiogram had evidence of abnormal electrocardiogram findings, especially pathological Q-waves, which may suggest a pre-clinical stage of disease.^47^

HCM is considered a worldwide disease, affecting different populations exposed to a large variety of environmental and geographic factors. Although the phenotypic expression of HCM does not show evidence of differences between the northern and southern hemisphere populations, it is still not clear whether they share the same genetic substrate. Molecular analysis of HCM patients from multiple geographic regions and ethnic groups would certainly contribute to the understanding of the complex characteristics of this condition. The genetic profile of HCM was defined based on investigations on unrelated populations in Europe and North America.^7-9,12-24^ More recently, the genetic-molecular analysis has been extended to cohorts in Asia,^25,26,28,30^ North Africa and Australia.^32^ On the other hand, data from populations from the south hemisphere are still scarce.^31-34^ In our country, molecular characteristics of HCM have been determined in patients from the southeast, north and northeast regions.^34^ The incorporation of more resolutive genetic tests to clinical practice would certainly expand their use in all continents.

In the present analysis, causing mutations were more frequent as compared with previous studies that included familial and sporadic HCM.^8,9,18,19,22-27^ This may be explained by the fact that our study group was composed of a well-characterized sample of individuals with HCM, even though it is known that the identification of mutations varies according with the study populations.^10^ All mutations detected in *MYH7* and *MYBPC3* were *missense* mutations. No mutation was found in the *TNNT2* gene, which may be explained by the lower prevalence of the gene in general population and the relatively older age of our sample, since the *TNNT2* gene is associated with an early onset and premature sudden death.^4,6,13^ In contrast to previous studies conducted on northern hemisphere populations,^7,8,11,22,23,27,48^ mutations in *MYH7* were more frequent than in *MYBPC3*. This characteristic was described in HCM patients from other regions in Brazil, and may represent a particularity of the disease in our country.^34^

Mutations in *MYH7* were found in 47% of patients that underwent genotyping and in 60% of the families. The screening of all coding regions of this gene identified the presence of four missense mutations in different protein domains. The mutation p.Ile263Thr has been previously reported in France,^7,22^ Portugal,^23^ and more recently, in other regions in Brazil.^34^ The mutation p.Ala797Thr has been previously reported in South Africa, with a possible founder effect, and in other cohorts in North America,^8^ North Africa,^27^ Europe^9,23,24^ Europe, as well as in Brazil.^34^ The mutation p.Met877Ile, novel, was mapped in one family with high degree of penetrance, affecting three of the four genotyped members of two generations. The mutation was associated with low to severe LVH, mid-ventricular and left ventricular outflow tract obstruction, and non-obstructive condition. The p.Glu1468Lys mutation, also considered novel, was identified in three unrelated families. Inter- and intrafamilial phenotypic variability was observed in obstructive and non-obstructive HCM, with mild to moderate LVH. This mutation was also related to mid-ventricular obstruction associated with late sudden death in one family member.

Missense mutations in the *MYBPC3* gene were found in 16% of patients and 20% of the families. The p.Arg495Gln mutation has been previously described in North America,^20^ Portugal,^23^ and reported as a frequent mutation in a cohort of HCM patients in Brazil.^34^ In this study, the mutation was identified in a family with history of premature sudden death and mild to massive LVH. The mutation p.Val896Met has been previously detected in European^7,23^ and South African^31^ cohorts. Two carriers of this mutation, a proband with moderate LVH and severe left ventricular outflow tract obstruction and a phenotype-negative young relative, also had the p.Glu1468Lys mutation in *MYH7*. This phenotype-negative individual showed pathological Q-waves in resting electrocardiogram. Double or compound heterozygosity have been usually identified in families with mutations in *MYBPC3*, representing 3-5% of HCM patients.^7,20^ Multiple mutations are commonly related to severe phenotypes and early onset disease, although varied degrees of LVH have been reported in these individuals.^34^

All mutations in the *MYH7* e *MYBPC3* genes showed marked intra- and interfamilial phenotypic variability, related to the degree of LVH. Phenotypic variability in carriers of the same mutation is considered a characteristic of HCM.^10^ The patterns of LVH and the age of disease onset may differ even between related subjects, since the phenotype is determined not only by the mutation *per se*, but also by the interaction of polymorphisms, modifying genes, and epigenetic and environmental factors.^6,10^ The meta-analysis of the genotype-phenotype associations in HCM showed that, due to the fact that many mutations are exclusive of a unique family, the studies conducted so far have not exhibited sufficient statistical power to reach definite conclusions.^48^ Nevertheless, some clinical variables may be related to the genotype, such as age, maximal wall thickness of LV and family history of HCM or sudden death. In our study group, there was a predominance of a family history of sudden death and the onset of the disease at an early age in carriers of mutation in the *MYBPC3* gene. These characteristics are different from those previously reported, but may represent a particularity of our group.

In 30% of the families, no causing mutation was identified and, in all these cases, there was no clinical history of HCM. The presence of a negative genetic test may result from the presence of mutations in unknown or unsequenced genes. LVH does not constitute a specific phenotype and may be identified in other heart conditions, seen as phenocopies of HCM. The detection of mutations in HCM-related sarcomeric genes has been associated with a family history or early onset of the disease, unfavorable prognosis and higher degree of LVH compared with patients with negative genetic tests.^9,25,27^ Recent data have indicated that this association occurs independently of the gene involved, although further studies to confirm this observation are still needed.^2^ In our study, a family history of HCM or sudden death was more frequent in genotype-positive individuals than in genotype-negative ones.

In this study, HCM causing mutations were identified in a considerable number of patients. However, the genetic profile of our population was not essentially different from that reported in cohorts of different ethnicities from other geographic regions, except for the fact that mutations in the *MYH7* gene were more frequent than in *MYBPC3*.

### Limitations of the study

The study consisted of the screening of the three most prevalent genes in HCM, but it did not include other genes of lower prevalence. Nevertheless, the analysis was restricted to a sample of unrelated families, registered in a unique tertiary care center located in the south of Brazil.

## Conclusions

In this first molecular-genetic analysis of a HCM cohort in the extreme south of Brazil, mutations were identified in 58% of consecutive individuals of unrelated families. The mutations affected predominantly the *MYH7* gene; this finding is different from that reported in northern hemisphere countries. Our study supports that mutations in *MYH7* and *MYBPC3* should be the first focus of molecular-genetic analysis in HCM, and that mutations in *TNNT2* have a low prevalence in Brazilian population. All mutations detected were missense mutations, whereas two mutations in *MYH7* had not been described before. The mutations affected different domains of the encoded proteins and determined variable phenotypic expressions. There was a relationship between a positive genetic test and a family history of HCM or sudden death. The predominance of mutations in the *MYH7* gene may be a characteristic of the local population.
